# Population preferences and attitudes towards COVID-19 vaccination: a cross-sectional study from Pakistan

**DOI:** 10.1186/s12889-021-11814-5

**Published:** 2021-09-26

**Authors:** Muhammad Junaid Tahir, Muhammad Saqlain, Waleed Tariq, Summaiya Waheed, Steven H. S. Tan, Sarim Irhas Nasir, Irfan Ullah, Ali Ahmed

**Affiliations:** 1 Ameer-ud-Din Medical College Affiliated with University of Health Sciences, Lahore, 54000 Pakistan; 2 Lahore General Hospital, Lahore, 54000 Pakistan; 3 Department of Pharmacy, Quaid-I-Azam University, Islamabad, 45320 Pakistan; 4 Dow Medical College, Dow University of Health Sciences, Karachi, Pakistan; 5 Newcastle University Medicine Malaysia, Nusajaya, Malaysia; 6 King Edward Medical University, Lahore, Pakistan; 7 Kabir Medical College, Gandhara University, Peshawar, Pakistan; 8 School of Pharmacy, Monash University, Jalan Lagoon Selatan, Bandar Sunway, 47500 Subang Jaya, Selangor Malaysia

**Keywords:** Vaccine, COVID-19, Pakistan, Myths, Rejection, Attitude, Barriers

## Abstract

**Background:**

While vaccine development is itself a challenge; ensuring optimal vaccine uptake at population level can present an even more significant challenge. Therefore, this study aimed to assess the Pakistani population’s attitude and preferences towards the Coronavirus disease 2019 (COVID-19) vaccine.

**Method:**

A cross-sectional study was carried out through an online self-administered questionnaire from 27 September 2020 to 11 October 2020. A total of 883 people responded to the survey. The questionnaire included the participants’ socio-demographic variables, attitudes, beliefs towards the COVID-19 vaccine and acceptance and rejection of vaccination, and reasons for them. Logistic regression analysis was used to analyze the predictors for vaccine acceptance and willingness to pay for the vaccine.

**Results:**

A majority (70.8%) of respondents will accept the COVID-19vaccine if available, and 66.8% showed a positive attitude towards vaccination. Monthly family income, education level, self-diagnosis of COVID-19 or a friend, family member, or colleague are significant factors influencing the acceptance of COVID-19 vaccination. The dogma of being naturally immune to COVID-19 was a key reason for the refusal of the vaccine. Less than half (48%) of those who refuse will vaccinate themselves if government officials have made it compulsory. A third (33.9%) of participants were willing to pay up to (7 USD) 1000 Pkr (Pakistani Rupees) for the vaccine.

**Conclusion:**

The population’s positive attitude should be improved by increasing awareness and eradicating false myths about vaccines through large-scale campaigns.

**Supplementary Information:**

The online version contains supplementary material available at 10.1186/s12889-021-11814-5.

## Background

Coronavirus disease 2019 (COVID-19) has posed a challenge to current health care, with 57.8 million cases and 1.3 million deaths reported worldwide as of November 2020 [1]. SARS-CoV2 virus, the causative agent for COVID-19 has a rapid spread rate. It is transmitted from person to person through a cough or sneezes aerosols, nasal discharge, saliva, urine & stool, and close contact with the infected person [2, 3]. The majority of COVID-19 infected people have mild or no symptoms, and they are a potential carrier of infection and may hasten disease transmission [4–6].

Vaccination has played an essential role in reducing the burden of diseases, disability, and deaths [7]. Every year, vaccination saves 4–5 million lives from deadly diseases [6]. This benefit has been used effectively and has resulted in numerous success stories against polio, tetanus, influenza, hepatitis B, diphtheria, pertussis, and MMR (Measles, Mumps, and Rubella). However, the vaccination coverage gap exists between countries, even within a country [8]. Vaccine hesitancy was regarded as one of the threats to global health in 2019 by the World Health Organization (WHO) [9]. Multiple reasons had led to vaccine hesitancy, such asmisguided safety concerns towards vaccinationled to measles, diphtheria, and pertussis outbreaks [7]. False religious beliefs and influential religious and political leaders significantly contributed to vaccination refusal [10]. Mongolia, Thailand, and Vietnam are known to have a high reluctance to vaccine due to strong religious beliefs [11]. There is also the role of personal or philosophical beliefs as active immunity acquired following natural infection is better than that acquired through vaccination, side effects of the vaccine outweigh its benefits, or a healthy lifestyle can prevent diseases [12].

Pakistani population immunization efforts are below the global vaccination standards. For example,vaccination coverage is reported to be 80% for Bacillus Calmette Guerin (BCG), 60% for polio, and 67% for measles [13]. This is attributable to logistical barriers, inefficiently trained health care personnel, poor parents awareness and education, political, religious, and business impacts on the marketing of vaccine products [13, 14]. Religious and political figures had played an important role in previous vaccination campaigns by promoting conspiracy theories. The unsuccessful story of polio eradication in Pakistan is also due to these notions [15]. The common factors for acceptance of COVID-19 vaccination include approval of its safety and effectiveness by the government, necessary recommendations by the employer, and cost-effectiveness [15]. Parents are also skeptical about the COVID-19 vaccine novelty [16]. Anti-vaccination groups are active against the vaccine, even denying the existence of COVID-19 [17]. The National Command and Control Center (NCOC) manages the COVID-19 vaccination program in Pakistan; frontline healthcare staff involved in patient care activities of those with confirmed and/or suspected COVID-19 will be prioritized for COVID-19 vaccination, followed by other healthcare workers and the general population [18].

Acceptance of the COVID-19 Vaccine is variable across the countries. For example 50% people in USA [19]. 93.3% in Indonesia [20], 62% in France, 80% in Denmark [21] and 59% in Italy [22] are willing to get vaccinated. Misleading narratives and religious conspiracy theories against the COVID-19 Vaccine in Pakistan could also affect the peoples’ decisions in taking the COVID-19 vaccine once available [23, 24], similar to how they contributed to polio’s refusal vaccination and hampered its eradication in Pakistan [11]. Hence, it is worth studying the Pakistani population’s response towards vaccination. Our study will also look into the Pakistani population’s responsiveness and willingness to receive the COVID-19 vaccine if it becomes widely available in the near future.

## Methods

### Ethical considerations

The study was an observational study that valued the anonymity and autonomy of the participant. The minimum age for participation in the survey was 18 years and above. Participants were allowed to withhold the completed form from the submission. The study did not contain any names or emails so that the participant could not be tracked. The study ensured that the privacy of each participant was adequately protected. Because no direct human or animal samples were obtained and only an academic questionnaire was used, and due to lockdowns and limited movement, universities were closed so study protocol was approved by the ethical review committee of tehsil headquarter hospital Samundri, Faisalabad (768/THQ/HR) and the study was carried out in accordance with the Helsinki Declaration.

### Study design and sample size calculation

A cross-sectional study design was adopted to collect data from the general population of Pakistan. Data was collected online, via a self-reported questionnaire, using Google Docs online, due to social distancing (physical distancing) and restricted movements and lockdowns. Given the high Internet use among people in Pakistan, a questionnaire link was distributed to respondents via Twitter, Messenger, emails, and WhatsApp groups.

According to the most recent Pakistani census, the country’s population is 207.7 million [25]. A minimum sample size of 601 was calculated by using an online Raosoft sample size calculator with 95% confidence interval, 50% proportion of the population, 4% margin of error, and 207.774 million total population. We continued collecting data from 27 September 2020 to 11 October 2020 to obtain more reliable and precise results, and we eventually received 883 responses to include in the study.

### Questionnaire development and data collection procedure

The questionnaire (attached as a supplementary file) was designed in two languages English and Urdu, after a thorough literature review [20, 26–28], to give freedom to study participants in whatever language they are comfortable participating in the study. After an initial draft of the questionnaire was designed, it was validated in 2 steps. Firstly, the study instrument was sent to researchers and professionals from the pharmacy and medical background to give their expert opinion concerning its simplicity, relativity, and importance. Secondly, a pilot study was conducted by selecting a small sample of participants (*n* = 60) who gave their opinions on making the questionnaire simpler and shorter. Modifications from the participants were considered and integrated into the questionnaire while ensuring its consistency with the published literature. The reliability coefficient was calculated using SPSS V.22, and the value of Cronbach’s alpha was 0.77. The data of the pilot study was not used for the final analysis. After a thorough discussion, the authors finalized the questionnaire and distributed it to the participants for their response.

The final data collection form included sections on socio-demographic information, previous COVID-19 infection, past vaccination history of diseases other than COVID-19, attitudes towards COVID-19 vaccination, acceptability of COVID-19 vaccination, reasons to vaccinate, and reasons to not vaccinate for COVID-19. Socio-demographic data included age, gender, marital status, residence (rural or urban), education, employment, and monthly family income. About COVID-19 infection, participants were asked about COVID-19 related symptoms, diagnosis by a healthcare professional, and any friend or family member diagnosed or died of COVID-19. The participants responded whether they would pay for the COVID-19 vaccine and how much Pkr (Pakistani Rupees) or USD (United States dollar) could pay for it. History of vaccination with Hepatitis B, seasonal Influenza, Tetanus, and Rabies was used to determine whether they had been vaccinated for currently available vaccines on the market. The attitude was assessed by asking 6 questions; each one was responded to as Yes (2), not sure (1), and No (0). The total score ranges from 0 to 12, with a score of 1–8 considered as a negative attitude and 9–12 (score greater than 75%) categorized as a positive attitude. In the last section, based on their acceptability of COVID-19 vaccination (Yes or No), participants responded about the reasons to get COVID-19 vaccination or not when it is available in the future. The data was collected by using a convenience sampling method through an online Google Document. At the start of the questionnaire, information about eligibility criteria and informed consent was clearly provided; those who agreed to provide informed consent were eligible to receive the full questionnaire for completion. Participants were not given any monetary benefits for participating in the study.

### Data analysis

Firstly, data were entered in Microsoft Excel and subsequently imported into SPSS V.22 for statistical analysis. Numerical variables were measured as mean and standard deviations, whereas categorical variables were expressed as frequencies and percentages. Inferential statistics were used, depending on the nature of the data and the variables. The participants’ predictors of acceptance and willingness to pay for the COVID-19 vaccine were determined using logistic regression analyses. In the first step, the association between dependent variables (vaccine acceptance and willingness to pay) and independent variables were analyzed in the univariate analysis. In the second step, all variables with *p* ≤ 0.2 in the first step were included in the multivariable analysis. The significance of crude odds ratio (COR) from univariate analyses and adjusted odds ratio (aOR) in multivariate analyses were assessed accompanied by 95% confidence intervals (CI). A *p*-value of < 0.05 was considered statistically significant.

## Results

### Sample characteristics

Out of 883 respondents, 55.6% (*n* = 491) were male, 49.3% (*n* = 435) were from the age group 21–30 years, with 81.3% (*n* = 718) were single. 80.2% (*n* = 708) of the respondents belonged to urban areas, and 59.6% (*n* = 526) had more than 13 years of formal education.

Only 8.7% (*n* = 77) had COVID-related symptoms, while 3.7% (*n* = 33) were diagnosed with COVID-19 by a health professional. The respondents had also reported that 42.2% (*n* = 461) and 26.7% (*n* = 236) had friends, family members, or colleagues diagnosed and died with COVID-19, respectively. Of all respondents, 9.1% (*n* = 80) had co-morbidities like diabetes, hypertension, heart disease, etc.

Only 42.1% (*n* = 372) of respondents had been vaccinated against influenza. 70.8% (*n* = 625) of the respondents were willing to get COVID-19 vaccination if it is available, while 29.2% (*n* = 258) were not. Out of 625 respondents, 70.7% (*n* = 442) were willing to pay for the COVID-19 vaccine if available, and 33.9% (*n* = 212) were willing to pay up to Pkr 1000 (7 USD) for the COVID-19 vaccine (Table 1).

**Table 1:** Demographics and COVID-19 related Characteristics of the Study Population

Variable	Frequency (n)	Percentages (%)
**Age**		
< 20	348	39.4
21–30	435	49.3
31–40	55	6.2
41–50	31	3.5
> 51	14	1.6
**Gender**		
Female	392	44.4
Male	491	55.6
**Marital Status**		
Married	165	18.7
Unmarried	718	81.3
**Residence**		
Rural	175	19.8
Urban	708	80.2
**Education**		
No formal education	21	2.4
≤ 10 years	45	5.1
11–12	291	33.0
≥ 13	526	59.6
**Employment**		
Employed	236	26.7
Non-Employed	647	73.3
**Monthly Family Income**		
< 25,000	175	19.8
25,000–50,000	415	47.0
> 50,000	293	32.2
**Do you have any COVID-related Symptoms?**		
Yes	77	8.7
No	701	79.4
Not sure	105	11.9
**Have you been diagnosed with COVID-19 by a health professional?**		
Yes	33	3.7
No	850	96.3
**Do you know any friend, family member, or colleague diagnosed with COVID-19?**		
Yes	461	42.2
No	422	47.8
**Do you know any friend, family member, or colleague died due to COVID-19?**		
No	647	73.3
Yes	236	26.7
**Do you have any chronic diseases? (Diabetes, Hypertension, Heart Disease, etc.)**		
No	803	90.9
Yes	80	9.1
**Do you plan to get COVID-19 vaccination, if it is available?**		
Yes	625	70.8
No	258	29.2
**Have you been vaccinated for the following in past?**		
Hepatitis B	395	44.73
Influenza	372	42.13
Tetanus	505	57.19
Rabies	95	10.76
**Will you pay for the COVID-19 vaccine?**		
Yes	442	70.72
No	183	29.28
**What maximum price will you pay for the COVID-19 vaccine?**		
Free	80	12.8
Up to Pkr 500	137	21.92
Up to Pkr 1000	212	33.92
Up to Pkr 5000	127	20.32
Up to Pkr 10,000	35	5.6
More than Pkr 10,000	34	5.44

### Attitude toward COVID-19 vaccination

Of all the respondents, 73.4% (*n* = 648) believed there would be a vaccine for COVID-19, and 58.0% (*n* = 512) believed that the COVID-19 vaccine would be safe. On the other hand, 62.7% (*n* = 554) and 53.1% (*n* = 469) believed that the COVID-19 vaccine would effectively prevent them from getting COVID-19. Due to the uncertainty of the vaccine as it is still under development and approval, 84.9% (*n* = 750) believed that more public awareness of the vaccine would be needed. As overall, 66.8% (*n* = 590) showed a positive attitude towards COVID-19 vaccination while 33.2% (*n* = 293) had a negative attitude (Table 2).

**Table 2:** Attitudes of Pakistani population towards COVID-19 vaccination

Answer the following questions.	Yes n (%)	No n (%)	Not sure n (%)
Do you believe that there will be a vaccine for COVID-19?	648 (73.4)	55 (6.2)	180 (20.4)
Do you believe that the COVID-19 vaccine will be safe?	512 (58.0)	62 (7.0)	309 (35.0)
Do you believe that the COVID-19 vaccine will be effective?	554 (62.7)	52 (5.9)	277 (31.4)
Do you believe that after vaccination you will be safe from COVID-19?	469 (53.1)	89 (10.1)	325 (36.8)
Do you believe that vaccine is the best way to be protected from COVID-19?	470 (53.2)	208 (23.6)	205 (23.2)
Do you believe that more public awareness is required about the COVID-19 vaccine?	750 (84.9)	66 (7.5)	67 (7.6)
**Overall Attitude towards COVID-19 vaccination**			
Negative Attitude	293 (33.2)		
Positive Attitude	590 (66.8)		

### Reasons for unwillingness to take the vaccine

In this study, 258 (29.2%) respondents reported that they do not wish to be vaccinated for COVID-19. The primary reasons for Unwillingness to take the vaccine include natural immunity against COVID-19 (48.4%), use of protective measures (39.9%), and concerns about side effects of the vaccine (32.2%). In this group, we further asked the circumstances; they would be willing to take the vaccine. A majority of the respondent (48.0%) will take the vaccine if the Government officials make the vaccination process compulsory forall citizens (Table 3).

**Table 3:** Reasons for not to vaccinate

Items	Responses n (%)
1. COVID-19 is not a serious disease.	46 (17.8)
2. COVID-19 is a conspiracy.	56 (21.71)
3. Vaccines have no role in disease prevention.	22 (8.53)
4. I would become infected due to vaccination.	23 (8.91)
5. I am worried about the side effects of vaccination.	83 (32.17)
6. I am naturally immune to COVID-19.	125 (48.44)
7. I am using protective measures against COVID-19.	103 (39.92)
8. I am afraid of needles.	23 (8.91)
9. I cannot afford the vaccine.	43 (16.67)
10. I am concerned if the vaccine is “halal”.	49 (18.99)
11. Vaccines are not properly stored in our country.	65 (25.19)
**Under what conditions, would you like to get the COVID-19 vaccine?**	
1. If Government officials make it compulsory.	124 (48.06)
2. If my doctor recommends it.	99 (38.37)
3. If my family or friends get vaccinated.	35 (13.56)
4. If it became compulsory for my job.	50 (19.38)
5. If there is a method other than injection.	22 (8.53)
6. I will not take it anyway.	55 (21.32)

### Reasons for willingness to take the vaccine

The respondents who were willing to take the vaccine were asked to state the reasons for vaccination. 81.6% (*n* = 510) wanted to protect themselves, and 72.6% (*n* = 454) wanted to protect the people around them from COVID-19 (Table 4).

**Table 4:** Reasons for Pakistani Population for Vaccination

Items	Response n (%)
1. To protect myselffrom COVID-19.	510 (81.6)
2. To protect people around me from COVID-19.	454 (72.64)
3. It would be made compulsory by health officials.	224 (35.84)
4. Vaccine is one of the best protection against diseases.	381 (60.96)
5. COVID-19 is a serious disease	310 (49.6)

### Predictors of acceptance for a COVID-19 vaccine

The results revealed that participants whose family income was Pkr 25,000–50,000 (USD 158–317) had more acceptability than participants with a family income of less than Pkr25000 (USD 158) (COR = 1.81, 95%CI = 0.75–2.63, *p* = 0.027; aOR = 1.73, 95%CI = 1.14–2.64, *p =* 0.010). It was also found that participants whose family, friends, or colleagues had died due to COVID-19 had more acceptance of the Vaccine (COR = 1.88, 95%CI = 1.32–2.69, *p = 0.001*; aOR = 1.74, 95%CI = 1.16–2.62, *p = 0.07*). A positive attitude regarding the COVID-19 vaccine was a prominent determinant of vaccine acceptability as participants with a positive attitude had 4.78 times higher acceptability than participants with a negative attitude (COR = 4.92, 95%CI = 3.61–6.72, *p =* < 0.001*;* aOR = 4.78, 95%CI: 3.47–6.59, *p =* < 0.001) (Table 5).

**Table 5:** Univariate and multivariate logistic regression analysis showing predictors of acceptance for a COVID-19 vaccine

Variable	Acceptability		Unadjusted		Adjusted	
	No	Yes	COR (95%CI)	P	aOR (95%CI)	P
**Age**						
< 20	98 (28.2)	250 (71.8)	1.00			
21–30	125 (28.7)	310 (71.3)	0.97 (0.71–1.33)	0.859		
31–40	19 (34.5)	36 (65.5)	0.74 (0.41–1.36)	0.334		
41–50	13 (41.9)	18 (58.1)	0..54 (0.26–1.15)	0.111		
> 51	3 (21.4)	11 (78.6)	1.44 (0.39–5.26)	0.584		
**Gender**						
Female	112 (43.4)	280 (44.8)	1.06 (0.79–1.42)	0.706		
Male	146 (56.6)	345 (55.2)	1.0			
**Marital Status?**						
Married	55 (33.3)	110 (66.7)	0.79 (0.55–1.13)	0.218		
Unmarried	203 (28.3)	515 (71.7)	1.0			
**Residence?**						
Urban	191 (27.0)	517 (73.0)	1.0		1.00	
Rural	67 (38.3)	108 (61.7)	0.59 (0.42–0.84)	0.003	0.87 (0.59–1.27)	0.48
**Education?**						
No formal education	6 (28.6)	15 (71.4)	1.00			
≤ 10 years	18 (40.0)	27 (60.0)	0.60 (0.19–1.84)	0.371		
11–12	95 (32.6)	196 (67.4)	0.83 (0.31–2.19)	0.700		
≥ 13	139 (26.4)	387 (73.6)	1.11 (0.42–2.93)	0.827		
**Employment?**						
Employed	75 (31.8)	161 (68.2)	0.88 (0.61–1.17)	0.312		
Non-Employed	183 (28.3)	464 (71.7)	1.0			
**Monthly Family Income?**						
< 25,000	65 (37.1)	110 (62.9)	1.00		1.00	
25,000–50,000	102 (34.8)	191 (65.2)	1.81 (0.75–2.63)	0.027	1.73 (1.14–2.64)	**0.010**
> 50,000	91 (21.9)	324 (78.1)	2.12 (1.43–3.09)	< 0.001	1.97 (0.63–2.98)	0.117
**Do you have any COVID-related Symptoms?**						
No	198 (28.2)	503 (71.8)	1.00			
Not sure	36 (34.3)	69 (65.7)	1.07 (0.46–1.63)	0.659		
Yes	24 (31.2)	53 (68.8)	1.15 (0.69–1.91)	0.590		
**Have you been diagnosed with COVID-19 by a health professional?**						
No	250 (29.4)	600 (70.6)	1.00			
Yes	8 (24.2)	25 (75.8)	1.32 (0.56–2.93)	0.523		
**Do you know any friend, family member, or colleague diagnosed with COVID-19?**						
No	145 (34.4)	277 (65.6)	1.00		1.00	
Yes	113 (24.5)	348 (75.5)	1.61 (1.20–2.16)	0.001	1.27 (0.91–1.79)	0.166
**Do you know any friend, family member, or colleague died due to COVID-19?**						
No	210 (32.5)	437 (67.5)	1.00		1.00	
Yes	48 (20.3)	188 (79.7)	1.88 (1.32–2.69)	0.001	1.74 (1.16–2.62)	**0.007**
**Do you have any chronic diseases? (Diabetes, Hypertension, Heart Disease, etc.)**						
No	230 (28.6)	573 (71.4)	1.00			
Yes	28 (35.0)	52 (65.0)	0.75 (0.46–1.21)	0.234		
**Overall Attitude towards COVID-19 Vaccine**						
Negative	152 (51.9)	141 (48.1)	1.00		1	
Positive	106 (18.0)	484 (82.0)	4.92 (3.61–6.72)	< 0.001	4.78 (3.47–6.59)	**< 0.001**

Variable with *P* < 0.2 were entered in multivariate logistic regression analysis. *COR* Crude odds Ratio, *aOR* adjusted odds ratio

*P-*value < 0.05 is considered statistically significant

### Predictors of willingness to pay for a COVID-19 vaccine

The formal education of more than 13 years(COR = 3.32, 95%CI = 1.18–9.40, *p = 0.024*;aOR = 4.37, 95%CI = 1.22–15.71, *p = 0.023*), a positive diagnosis of COVID-19 (COR = 10.45, 95%CI = 1.40–77.89, *p = 0.022*;aOR = 13.05, 95%CI = 1.49–93.97, *p = 0.020*), and a positive attitude towards the COVID-19 vaccine (COR = 2.26, 95%CI = 1.53–3.35, *p =* < 0.001;aOR = 2.23, 95%CI = 1.48–3.36, *p =* < 0.001) had a statistically significant association with willingness to pay for the vaccine (Table 6).

**Table 6:** Univariate and multivariate logistic regression analysis showing the predictors of willingness to pay for a COVID-19 vaccine

Variable	Willing to Pay		Unadjusted		Adjusted	
	No	Yes	COR (95%CI)	P	aOR (95%CI)	P
**Age**						
< 20	78 (31.2)	172 (68.8)	1.00			
21–30	81 (26.1)	229 (73.9)	1.28 (0.89–1.85)	0.286		
31–40	12 (33.3)	24 (66.7)	0.91 (0.43–1.91)	0.797		
41–50	7 (38.9)	11 (61.1)	0.71 (0.27–1.90).	0.500		
> 51	5 (45.5)	6 (54.5)	0.54 (0.16–1.84)	0.327		
**Gender**						
Female	81 (28.9)	199 (71.1)	1.03 (0.73–1.45)	0.862		
Male	102 (29.6)	243 (70.4)	1.00			
**Marital Status?**						
Married	34 (30.9)	76 (69.1)	0.91 (0,58–1.43)	0.679		
Unmarried	149 (28.9)	366 (71.1)	1.00			
**Residence?**						
Urban	145 (28.0)	372 (72.0)	1.39 (0.89–2.16)	0.239		
Rural	38 (35.2)	70 (64.8)	1.00			
**Education?**						
No formal education	8 (53.3)	7 (46.7)	1.00		1.00	
≤ 10 years	16 (59.3)	11 (40.7)	0.78 (0.22–2.80)	0.110	1.16 (0.27–5.06)	0.837
11–12	60 (30.6)	136 (69.4)	2.59 (0.89–7.46)	0.078	3.32 (0.92–12.09)	0.066
≥ 13	99 (25.6)	288 (74.4)	3.32 (1.18–9.40)	0.024	4.37 (1.22–15.71)	**0.023**
**Employment?**						
Employed	38 (23.6)	123 (76.4)	1.47 (0.97–2.24)	0.067	1.14 (0.72–2.04)	0.176
Non-Employed	145 (31.3)	319 (68.8)	1.00		1.00	
**Monthly Family Income?**						
< 25,000	35 (31.8)	75 (68.2)	1.00		1.00	
25,000–50,000	78 (24.1)	246 (75.9)	1.47 (0.92–2.37)	0.111	1.21 (0.71–2.04)	0.483
> 50,000			1.81 (1.49–2.33)	0.197	1.72 (1.42–2.24)	0.247
**Do you have any COVID-related Symptoms?**						
No	155 (30.8)	348 (69.2)	1.00		1.00	
Not sure	19 (27.5)	50 (72.5)	1.17 (0.67–2.05)	0.179	1.41 (0.76–2.61)	0.270
Yes	9 (17.0)	44 (83.0)	2.18 (1.04–4.57)	0.040	1.43 (0.64–3.17)	0.379
**Have you been diagnosed with COVID-19 by a health professional?**						
No	182 (30.3)	418 (69.7)	1.00		1.00	
Yes	1 (4.0)	24 (96.0)	10.45 (1.40–77.89)	0.022	13.05 (1.49–93.97)	**0.020**
**Do you know any friend, family member, or colleague diagnosed with COVID-19?**						
No	94 (33.9)	183 (66.1)	1.00		1.00	
Yes	89 (25.6)	259 (74.4)	1.45 (1.06–2.11)	0.023	1.05 (0.69–1.59)	0.204
**Do you know any friend, family member, or colleague died due to COVID-19?**						
No	136 (31.1)	301 (68.9)	1.00		1.00	
Yes	47 (25.0)	141 (75.0)	1.35 (0.92–1.99)	0.124	1.22 (0.78–1.90)	0.376
**Do you have any chronic diseases? (Diabetes, Hypertension, Heart Disease, etc.)**						
No	168 (29.3)	405 (70.7)	1.00			
Yes	15 (28.8)	37 (71.2)	1.02 (0.56–1.91)	0.943		
**Overall Attitude towards COVID-19 vaccine**						
Negative	61 (43.3)	80 (56.7)	1.00		1.00	
Positive	122 (25.2)	362 (74.8)	2.26 (1.53–3.35)	< 0.001	2.23 (1.48–3.36)	**< 0.001**

Variable with *P* < 0.2 were entered in multivariate logistic regression analysis. *COR* Crude odds Ratio, *aOR* adjusted odds ratio

*P-*value < 0.05 is considered statistically significant

## Discussion

The lack of effective treatment for COVID-19 and the contagious nature have created a need for active immunization through immediate vaccination [29]. Moreover, countries with fragile economies cannot benefit from an overly extensive lockdown, therefore, vaccination is particularly vital in such settings [24]. While vaccine production is itself a challenge, convincing people to vaccinate is another big problem [30]. Vaccine hesitancy, which is quite prevalent in South Asia, including many parts of Pakistan, is when people pursue the pros and cons of a vaccine, leading to either complete rejection or delayed acceptance of the vaccine despite its easy provision [24, 31]. Multiple studies have been conducted to assess the public response regarding the COVID-19 vaccine [20, 21, 30, 32, 33]. With its suboptimal healthcare facilities, high population density, and poor hygiene practices, Pakistan can suffer untold and lasting consequences as a result of the ongoing pandemic, especially if there is significant resistance to COVID-19 vaccination [24]. In a country struggling to eradicate diseases such as polio and measles [5], resistance to vaccination against a new disease like COVID-19 can be anticipated. Therefore, it is important to study the public response to vaccination against vaccination COVID-19.

Our findings indicate that a majority (70.8%) of the participants were willing to be vaccinated against COVID-19.Similar results were seen from certain European countries where by willingness to take the vaccine was found to be 62% in France, 80% in Denmark, and the UK [21]. A study from the USA showed 57.6% intended to take the vaccine [33]. An Indonesian study showed 93.3% and 67 of participants would want to get vaccinated provided the effectiveness was 95 and 50%, respectively [20].

In our study, 66.8% of respondents reflected a positive attitude towards the COVID-19vaccination.This positive attitude towards the COVID-19 vaccination was further found to have a significant relation with vaccine acceptance. The participants witha monthly family income of more than Pkr50000 (>USD 317) had a better COVID-19 vaccine acceptance rate (78%). Likewise, lower-income groups were less willing to take the vaccine [19, 32]. Financial constraints are one of the identified reasons for reduced vaccination uptake [34]. Pakistan is a developing country with a poverty rate of 75.4%, as reported in 2015 [35]. Thus, it is understandable why family income plays a crucial role in vaccine acceptance.

Another predictor of acceptance for the vaccine was the knowledge of the death of any friend, family member, or colleague from COVID-19. The emergence of the COVID-19 pandemic has given rise to conspiracy thinking of a great magnitude. Since people have their false ideation, they are less likely to comply with necessary measures to combat the disease outbreak [36]. Conspiracies pertaining to the COVID-19 vaccine are widespread in Pakistan, channeled through social media platforms [24]. The influential and authoritative personalities in country have dubious remarks against vaccination, claiming virus to be an illusion against Islamic nations, empowering Jews to rule the world, implantation of nano-chips inside people to have a full surveillance of them through 5G towers, and false beliefs of deliberate creation of the virus for the sake of global spread presents a worrisome challenge, as it can fuel the emerging doubts amongst people who will then resist the idea of vaccination after hearing such statements [17, 24, 37]. Witnessing a close person infected with COVID-19 might aid in removing the blindfold of such conspiracies, thus encouraging people to adhere to the experts’ recommendations. This can be an underlying explanation as to why people who were acquainted with deaths were more likely to get themselves vaccinated. However, this finding contrasts with a study conducted in the United States by Pogue et al. that found no link between vaccine acceptance and how close the participants were with the diagnosed patient, nor any relation with the severity of their disease [32]. It could be because 88.7% of the participants in our study were less than 30 years of age and only 32.7% in the study by Pogue et al. [32]. Young people (as in our study) are usually more emotionally derived and have more psychological influence, hence easy to remove conspiracies from them, and likely to get themselves vaccinated. The other reason could be the small sample size in their study.

A positive attitude towards the COVID-19 vaccine was found to correlate with the willingness to pay for the immunization significantly. Among the predictors of willingness to pay for a COVID-19 vaccine, the formal education of more than 13 years was significant. It can be justified that higher education creates better job opportunities and thus leads to improved financial outcomes. Furthermore, higher education leads to increased awareness, rejection of myths and conspiracy beliefs. Public awareness regarding infectious diseases hasshown to increase the confidence in vaccines [24]. A person is more likely to understand the gravity of the situation and will be willing to financially spend on measures taken to fight the crisis to minimize the potential for harm. Hesitancy to take the vaccinecan also lead to an unwillingnessto pay for the vaccine [38]. Our result concursthat lower education status was the leading predictor of reluctance to take vaccines [33].

Another predictor that had a significant relationship with the willingness to pay for the vaccine was being diagnosed with COVID-19 by a health professional. Previous studies observed people who were vulnerable to infections were more eager to get vaccinated [20]. Being infected once and diagnosed by a healthcare professional can create fear of experiencing the same condition again and create a more cautious behavior in the future for maintaining one’s well-being. This might influence a previously affected patient to be more willing to pay for a vaccine [39]. This finding is, however, in contrast, to study by Sherman et al.*,* which was also An online questionnaire-based cross-sectional study like ours but had a larger sample size from a developed country (UK), and they found no association between vulnerability to COVID-19 and intention for vaccination [40] but is similar toWang et al.that people with a high or very high risk of infection were more inclined towards COVID-19 vaccination [41].

Regarding the reasons for vaccination, people wanted to protect themselves (81.6%) and the people around them (72.6%) from COVID-19. A UK-based study observed a similar result where being a potential source of infection to others was related to the intention of getting vaccinated [40]. The most common reason for vaccine denial was having natural immunity to COVID-19 (48.4%). This is concerning, since there have been confirmed reinfection with SARS-CoV2 virus and reported disease activation cases [42]. Since the virus is a new strain and under constant study, it is difficult to comment on the type of immunity that follows after infection.. Thus, lifelong immunity should not be considered a post-COVID-19 infection. Therefore, instead of taking the risk especially considering the deadlier and unknown nature of COVID-19 one should opt for a proven successful method of controlling infectious diseases, that is, a vaccine [41].

About 48% of participants responded that they would get vaccinated if Government officials made it compulsory while 38% said they would get the vaccine if their doctor recommended it. The results are supported by previous studies that noticed a parallel link between recommendations by a healthcare provider and inclination towards vaccination [19]. In collaboration with government, religious, and media personalities, healthcare providers can play a pivotal role in the community, for they are trusted upon and sought for advice by the public. With a profound knowledge of the vaccine’s efficacy and safety profile, healthcare professionals can confidently convince them to get vaccinated [43].

The significance of vaccination can be acknowledged by past evidence,which demonstrates that vaccination protects not only those who are vaccinated but also the unvaccinated through herd immunity [33]. However, to have the desired level of immunity in society, individuals should be willing to get a vaccination in the first place [21]. Thus, play their role in achieving the percentage of the vaccinated population required for herd immunity. Immunization campaigns in Pakistan are regulated by the Expanded Program on Immunization (EPI) in collaboration with WHO. Owing to its close interaction with the general population, EPI staff can help correct the myths floating around regarding COVID-19 [24]. To encourage people towards vaccination against COVID-19,the responsibility falls upon the Pakistani media not to broadcast content that fuel the conspiracies against COVID-19. The Pakistan Electronic Media Regulatory Authority (PEMRA), in association with the Ministry of Information, is responsible for controlling media content, therefore it should ensure false news, and suspicious remarks related to COVID-19 are not aired, and if channels promote misinformation, they should be held accountable [31]. A teleportal should be set up where the public can direct their queries and fears, and experts can respond and comment on vaccine safety [41]. As it has been shown, public-awareness campaigns have a positive relation with vaccine confidence [44]. Mass awareness campaigns in the country should be channeled through social media platforms, television, radio, newspapers, billboards, etc. Special emphasis on the need for vaccination should be made by highlighting the past successes achieved through vaccines. Consideration for the provision of financial incentives for vaccination should be considered [41]. There is a need to address religious concerns and conspiracy theories attributable to COVID-19 vaccination [45]. Conspiracies often attributed to religion are promoted, as was seen with the case of polio vaccines, where false beliefs regarding vaccine containing pig or monkey derivatives were articulated [24]. Involving religious scholars and having them address the public regarding the importance of vaccination according to Islamic Sharia law, will curb the doubts among the public [46]. To reduce the spread of false information, effective investigation of the source of spread, mode of spread, and its impact on the public is required. To do so, researchers and public health educators should develop a system of approach to containthe spread of incorrect information and conspiracies [17, 47]. Upon the vaccine’s arrival, a nationwide immunization program should be devised and measures should be taken to ensure smooth and sufficient immunization coverage to reach herd immunity [41, 48].

### Limitations

Our study has some limitations. Firstly, the distribution of the questionnaire was carried out online and not physically, which may precipitate a selection bias since we might have missed out on responses from certain population segments,which could have affected our results otherwise. Secondly, the study was based on the assumption of a hypothetical vaccine. The real-life scenario could be different as the real vaccine’s effectiveness, side effect profile, costs, and availability may differ and influence public response. To adjust bias, adjusted regression models were applied. We didn’t set any particular acceptable response rate. We calculated the required minimum obligator sample size based on at least 50% of the population’s response.

## Conclusion

The factors against the acceptance of the COVID-19 vaccine include low levels of formal education and family income. People are willing to vaccinate themselves for their protection. The challenges anticipated in a successful vaccination program should be overcome by a collective approach of health professionals, social workers, the government, and the general population. Healthcare professionals should highlight the significance and the government should bear the expenses of vaccines.

## Supplementary Information

**Additional file 1.**

## Data Availability

Data is available from the corresponding author on a reasonable request.

## Abbreviations

MMR: Measles, Mumps, and Rubella
USA: United States of America
WHO: World Health Organization
Pkr: Pakistani rupees
USD: United States dollar
EPI: Expanded Program on Immunization
PEMRA: Pakistan Electronic Media Regulatory Authority
