# Supplementary material for: Population preferences and attitudes towards COVID-19 vaccination: a cross-sectional study from Pakistan
Source: BMC Public Health. 2021 Sep 26;21:1759. doi: 10.1186/s12889-021-11814-5 (PMC8474768; doi:10.1186/s12889-021-11814-5)
Supplement: Supplementary file 1 — Additional file 1. [file 12889_2021_11814_MOESM1_ESM.docx]

**Population Preferences and Attitudes Towards COVID-19 Vaccination: A Cross-Sectional Study from Pakistan**

Biodata

1. Age ( In years )
2. Gender (Male, Female)
3. Marital status (Married, Unmarried)
4. Residence (Urban, Rural)
5. Education (No formal education, Primary, Middle, Matric, Intermediate, Graduation, Post-graduation)
6. Employment (Employed, Unemployed)
7. Monthly family income (< 25000, 25000-50000, >50000 )
8. Do you have any COVID-19 related symptoms? (Yes, No, Not sure)
9. Have you been diagnosed with COVID-19 by a health professional? (Yes, No)
10. Do you know any friend, family member or colleague diagnosed with COVID-19? (Yes, No)
11. Do you know any friend, family member, or colleague died due to COVID-19? (Yes, No) (Yes, No)
12. Do you have any chronic disease? (Diabetes, Hypertension, Heart Disease, etc.) (Yes, No)
13. Have you been vaccinated for the following in past? (Check for all that apply)

.Hepatitis B .Seasonal influenza .Tetanus . Rabies

Attitude toward COVID-19 Vaccination

| Answer the following questions. | Yes | No | Not sure |
| --- | --- | --- | --- |
| 1. Do you believe that there will be a vaccine for COVID-19? |  |  |  |
| 1. Do you believe that COVID-19 vaccine will be safe? |  |  |  |
| 1. Do you believe that COVID-19 vaccine will be effective? |  |  |  |
| 1. Do you believe that after vaccination you will be safe from COVID-19? |  |  |  |
| 1. Do you believe that vaccine is the best way to be protected from COVID-19? |  |  |  |
| 1. Do you believe that more public awareness is required about COVID-19 vaccine? |  |  |  |

Availability of Vaccine

1. Do you plan to get COVID-19 vaccination, if it is available?

.Yes .No

Reasons for Not vaccination

| Check all the reasons for not getting the COVID-19 vaccine? | Check all that apply |
| --- | --- |
| 1. COVID-19 is not a serious disease. |  |
| 2. COVID-19 is a conspiracy. |  |
| 3. Vaccines have no role in disease prevention. |  |
| 4. I would become infected due to vaccination. |  |
| 5. I am worried about side effects of vaccination. |  |
| 6. I am naturally immune to COVID-19. |  |
| 7. I am using protective measures against COVID-19. |  |
| 8. I am afraid of needles. |  |
| 9. I cannot afford vaccine. |  |
| 10. I am concerned if the vaccine is "halal”. |  |
| 11. Vaccines are not properly stored in our country. |  |
| 12.Other |  |

| Under what conditions, would you like to get COVID-19 vaccine? | Check all that apply |
| --- | --- |
| 1. If Government officials make it compulsory. |  |
| 2. If my doctor recommend it. |  |
| 3. If my family or friends get vaccinated. |  |
| 4. If it become compulsory for my job. |  |
| 5. If there is a method other than injection. |  |
| 6. I will not take it anyway. |  |
| 7.Other |  |

Reasons for Vaccination

| Check all the reasons for getting the COVID-19 vaccine? | Check all that apply |
| --- | --- |
| 1. To protect myself from COVID-19. |  |
| 2. To protect people around me from COVID-19. |  |
| 3. It would be made compulsory by health officials. |  |
| 4. Vaccine is one of the best protection against diseases. |  |
| 5. COVID-19 is a serious disease |  |
| 6.Other |  |

1. Will you pay for the COVID-19 vaccine if it is not free?

. Yes .No .Not sure

2.What maximum price will you pay for COVID-19 vaccine?

.Free .Up to Rs.500 .Up to Rs.1000 .Up to Rs.5000

.Up to Rs.10000 . More than Rs.10000
